# Effects of prophylactic dexamethasone on postoperative nausea and vomiting in scoliosis correction surgery: a double-blind, randomized, placebo-controlled clinical trial

**DOI:** 10.1038/s41598-019-38764-8

**Published:** 2019-02-14

**Authors:** Rie Wakamiya, Hiroyuki Seki, Satoshi Ideno, Naho Ihara, Rie Minoshima, Kota Watanabe, Yasunori Sato, Hiroshi Morisaki

**Affiliations:** 1 Department of Anesthesiology, Keio University School of Medicine, Tokyo, Japan; 2 Department of Orthopaedic Surgery, Keio University School of Medicine, Tokyo, Japan; 3 Department of Preventive Medicine and Public Health, Keio University School of Medicine, Tokyo, Japan

## Abstract

Dexamethasone is widely used for postoperative nausea and vomiting (PONV) prophylaxis, but its effect on PONV prevention in paediatric patients is validated only in short minor surgical procedures. In this study, we aimed to determine whether a single dose of dexamethasone reduces PONV in highly invasive surgeries that require opioid-based postoperative analgesia. One hundred adolescents undergoing scoliosis correction surgery were randomized to receive intravenous dexamethasone 0.15 mg/kg (dexamethasone group) or saline (control group) at induction of anaesthesia. The primary outcome was the incidence of PONV in the 72 h postoperatively. Data for 98 patients were available for analysis. The 72-h incidence of PONV was significantly lower in the dexamethasone group than in the control group (62.5% vs 84.0%; RR 0.74, 95% CI 0.58–0.96, P = 0.02). During the first and second 24-h postoperative intervals, fewer patients in the dexamethasone group received rescue antiemetics. Visual analogue scale scores for nausea and pain were lower in the dexamethasone group than in the control group during the first 24 h postoperatively. Dexamethasone did not increase the number of adverse events. The results of this study showed that a single dose of dexamethasone was effective for reducing PONV after paediatric scoliosis correction surgery.

## Introduction

Scoliosis correction surgery has been described as the most invasive orthopaedic surgery performed in young persons^1^. This surgery is associated with severe postoperative pain that requires advanced pain management, which is typically opioid-based patient-controlled analgesia (PCA). However, the management of opioid-related complications, in particular postoperative nausea and vomiting (PONV), is still inadequate in the majority of patients^1^. Serious outcomes from anaesthesia are rare, but PONV is a major concern in surgical patients^2^. PONV can impair patient satisfaction, delay postoperative recovery, and increase medical costs^3^. PONV affects approximately one-third of surgical patients and up to 70% of high-risk patients^4^. For patients at a high risk of PONV, prophylactic use of antiemetics including corticosteroids is recommended in international consensus guidelines^5^. However, the evidence for prophylactic use of dexamethasone in paediatric patients is based only on minor surgical procedures, such as tonsillectomy and strabismus surgery^6,7^, in which PONV may be induced by volatile anaesthetics or intraoperative opioids. Although a multicentre, multinational survey demonstrated that the use of dexamethasone for PONV prophylaxis is common practice in paediatric scoliosis surgery^8^, the effect of dexamethasone on PONV prevention has not been validated for highly-invasive surgical procedures that require opioid-based PCA for postoperative analgesia, in which PONV can be prolonged by opioids.

The aim of this study was to determine whether dexamethasone reduces PONV in children and adolescents undergoing highly invasive surgery, such as posterior correction and spinal fusion surgery for adolescent idiopathic scoliosis (AIS).

## Materials and Methods

### Patient recruitment and randomization

This randomized, single-centre, double-blind, prospective, placebo-controlled clinical trial was approved by the ethical committee at Keio University School of Medicine on 23 February 2015 (protocol number 20140395). The study was registered on the University Hospital Medical Information Network (UMIN) Clinical Trials Registry on 19 March 2015 (UMIN000016847). The study was conducted in accordance with the ethical standards of the Declaration of Helsinki of 1975.

Patients aged 10–19 years with a diagnosis of AIS and scheduled for posterior correction and fusion surgery at Keio University Hospital from May 2015 onwards were eligible for participation. The exclusion criteria were use of corticosteroids within the month before surgery, use of an antiemetic in the 24 h before surgery, and a contraindication to the study drug. Written informed consent was obtained from the parents of the patients along with assent from the patients.

Before surgery, the patients were provided with instructions regarding use of the PCA device and the visual analogue scale (VAS) for nausea and pain (0–100 mm).

On the day of surgery, the patients were randomly assigned 1:1 to either of two study groups using a computer-generated random number table. Randomization was performed by an anaesthesiologist who was not involved in the trial. Participants and their parents, the surgeon, anaesthesiologists, nurses, and the investigator following the participant postoperatively were blinded to study group allocation.

### Study intervention

All patients received intravenous propofol 2.5 mg/kg, fentanyl 4 μg/kg, and rocuronium 0.6 mg/kg for induction of anaesthesia. At this time, the patients also received intravenous dexamethasone 0.15 mg/kg in 5 ml of 0.9% normal saline (dexamethasone group; n = 50) or volume-equivalent 0.9% normal saline (control group; n = 50). The study drugs were prepared by a pharmacist who was not involved in the study. The patients, parents, and health care providers including the anaesthesiologists and nurses in the operating room, intensive care unit and on the floor remained unaware of the group assignment for an individual subject. After tracheal intubation, anaesthesia was maintained with propofol (adjusted to maintain a bispectral index of 40–60), intermittent administration of fentanyl, and infusion of remifentanil. Motor-evoked potentials were monitored after administration of sugammadex with the patient in the prone position. After emergence from anaesthesia, the trachea was extubated, and the patient was transferred to the intensive care unit.

Postoperative analgesia consisted of intravenous PCA with fentanyl (0.2 μg/kg/h as a background infusion and 0.4 μg/kg as a bolus dose, with a lockout interval of 10 min), infusion of ketamine 0.1 mg/kg/h, and administration of a 25-mg diclofenac sodium suppository every 6 h. In addition, IV flurbiprofen 50 mg was administered upon patients’ request. Metoclopramide 10 mg was administered intravenously to treat nausea on patient request. Intravenous PCA was continued for at least 3 days postoperatively unless otherwise specified.

At 24, 48, and 72 h after surgery, all patients were asked by trained ward nurses blinded to study group assignment to complete a VAS sheet describing the worst levels of nausea and pain experienced during the preceding interval.

### Outcome measures

The primary outcome measure was the incidence of PONV in the 72 h after surgery. Nausea was defined as a subjective feeling of a desire to vomit without the presence of expulsive muscular movements. Vomiting was defined as the involuntary, forceful expulsion of the contents of stomach. The patient was considered to have nausea when the VAS score for nausea was more than 0 and to have PONV if nausea or vomiting had occurred or rescue metoclopramide had been administered. Retching was included in vomiting.

The secondary outcomes included the incidence of PONV, vomiting, use of rescue metoclopramide, VAS scores for nausea and pain in the 0–24, 24–48, and 48–72 h after surgery, number of PCA doses requested by the patient and total amount of fentanyl administered in the 72 h postoperatively, amount of blood loss in the 24 hours after surgery, and incidence of surgical site infection in the month following surgery.

### Power calculation and statistical analysis

A power analysis was performed using a power of 80% and an α of 0.05 (two-sided). Our retrospective observations showed that 26 (79%) of 33 patients experienced PONV within 72 h after surgery. Thus, we assumed that the incidence of PONV in the 72 h after surgery in the control group would be approximately 80%. We considered that a 30% reduction in incidence of PONV would be clinically relevant. The power analysis showed that 46 patients were needed in each study group. Fifty patients were enrolled in each group to allow for possible dropouts.

The data were analysed by a statistician who was not involved in the study or data collection, based on the intention-to-treat population, i.e., all patients who were randomized, received the study drug, and underwent surgery. Categorical data are presented as frequencies and continuous data are summarised as the mean (standard deviation or standard error) or median [interquartile range]. Continuous parametric and non-parametric data were compared using the Student’s *t*-test and Mann-Whitney *U* test, respectively. Categorical data were compared using Fisher’s exact test. For sensitivity analysis, the incidence of PONV, vomiting, use of rescue metoclopramide, VAS scores for nausea and pain at each time-point were estimated by the generalised linear mixed model (GLMM), to obtain point estimates and 95% confidence limits. The correlation structure was assumed as Toeplitz, autoregressive, or compound-symmetry structures were used in order if convergence was not obtained. The statistical analyses were performed using SPSS software version 22 (IBM Corp., Armonk, NY, USA) and SAS software version 9.4 (SAS Institute Inc., Cary, NC, USA). All tests were 2-sided. A P-value of <0.05 was considered statistically significant.

## Results

**Figure 1:**
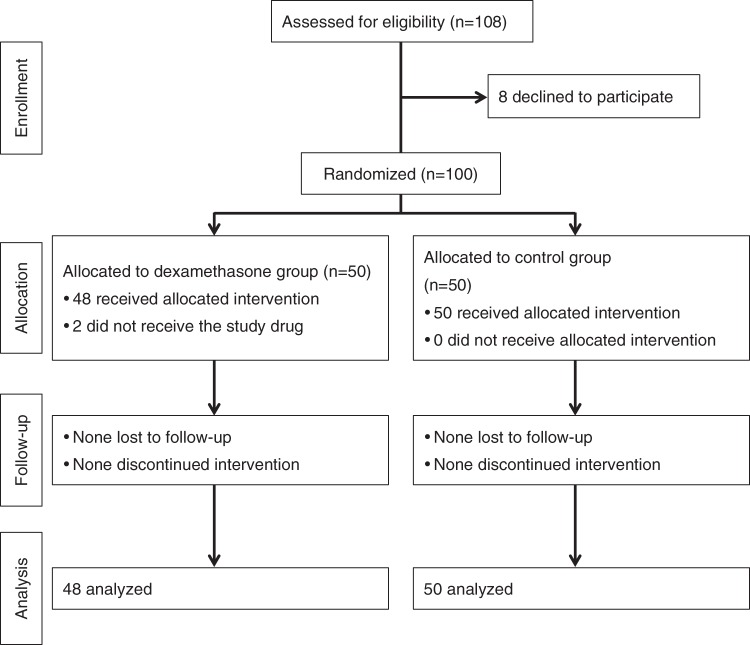
Flow chart of the study patients.

One hundred and eight patients underwent scoliosis correction surgery at our institution from May 2015 to August 2017. The first participant was enrolled on May 18, 2015. Eight patients refused to participate in the study, leaving 100 patients who underwent surgery for enrolment. Two patients in the dexamethasone group were subsequently excluded because of a protocol violation (the study drugs were not administered), leaving data for 98 patients (48 in the dexamethasone group and 50 in the control group) available for analysis (Fig. 1). The patient characteristics and intraoperative variables are shown in Table 1. Although two patients in the control group had a history of gastrointestinal surgery (appendectomy and intussusception surgery), no patients had gastrointestinal problems at the time of our study.

**Table 1:** Patient characteristics and intraoperative variables.

Variable	Control (n = 50)	Dexamethasone (n = 48)	Difference in mean (95% CI)	P-value
Age, y	14.4 (1.7)	14.6 (2.2)	0.22 (−0.55–1.00)	0.56^a^
Male: Female	3: 47	6: 42		0.3^a^
Height, cm	157.7 (6.4)	158.0 (6.4)	0.55 (−2.01–3.12)	0.67^a^
Weight, kg	45.0 (6.8)	47.1 (7.2)	2.12 (−0.68–4.94)	0.14^a^
Cobb angle	55.0 (13.5)	54.9 (10.3)		0.97^a^
Lenke type, n				0.52^c^
1	20	23		
2	8	11		
3	2	1		
5	14	7		
6	6	6		
Curve type, n				0.39^c^
Single thoracic	20	23		
Double thoracic	8	11		
Double major	8	7		
Single lumber	14	7		
Number of levels fused	7 [5–10]	9 [7–11]		0.10^b^
Duration of surgery, min	131.0 (43.4)	128.3 (31.7)	2.7 (−12.6–18.0)	0.72^a^
Duration of anesthesia, min	204.2 (46.4)	201.4 (38.8)	2.6 (−14.6–19.8)	0.76^a^
Intraoperative fentanyl, μg/kg	10.9 (2.0)	11.3 (3.1)	−0.4 (−1.5–0.6)	0.42^a^
Intraoperative remifentanil, μg/kg	47.9 (16.1)	46.3 (12.9)	1.7 (−4.2–7.5)	0.57^a^
Intraoperative blood loss, ml	276.1 (170.2)	323.8 (204.5)	−47.6 (−123.0–27.7)	0.21^a^

^a^The Student’s t-test, ^b^Mann-Whitney *U* test and ^c^Fisher’s exact test were used. Values are shown as the mean (standard deviation), ratio or median [interquartile range].

The incidence of PONV in the 72 h after surgery was significantly lower in the dexamethasone group than in the control group (62.5% vs 84.0%; number needed to treat 4.7, relative risk (RR) 0.74, 95% confidence interval (CI) 0.58–0.96, P = 0.02; Table 2). The frequency of PONV decreased with time, but remained high even after 48 h, particularly in the control group. Significantly fewer patients in the dexamethasone group experienced PONV during the first and second 24-h intervals than those in the control group (RR 0.27, 95% CI 0.11–0.67 and RR 0.40, 95% CI 0.18–0.91; P < 0.01 and P = 0.03, respectively). There were no significant differences between the study groups in the incidence of PONV during the 48–72 h after surgery (29.6% vs 48.0%, RR 0.54, 95% CI 0.24–1.24, P = 0.15).

**Table 2:** Primary and secondary outcomes.

	Dexamethasone (n = 48)	Control (n = 50)	Between- group difference (95% CI)	RR (95% CI)	P-value
**Primary outcome**					
PONV 0–72 h, % (Number of patients)					
	62.5 (30)	84.0 (42)		0.74 (0.58–0.96)	0.02^b^
**Secondary outcomes**					
PONV, % (Number of patients)					
0–24 h	41.7 (25)	80.0 (40)		0.27 (0.11–0.67)	<0.01^d^
24–48 h	33.3 (20)	64.0 (32)		0.40 (0.18–0.91)	0.03^d^
48–72 h	29.6 (16)	48.0 (24)		0.54 (0.24–1.24)	0.15^d^
Patients with data	48	50			
VAS score for nausea, mm, estimation (SE)					
0–24 h	24.8 (8.6)	41.6 (8.5)	16.8 (4.6–29.0)		<0.01^d^
24–48 h	19.4 (8.6)	26.4 (8.5)	7.0 (−5.2–19.1)		0.26^d^
48–72 h	17.3 (8.6)	12.9 (8.5)	−4.4 (−16.6–7.7)		0.48^d^
Patients with data	48	50			—
Vomiting, % (Number of patients)					
0–24 h	27.1 (13)	32.0 (16)		1.17 (0.52–2.61)	0.70^d^
24–48 h	10.4 (5)	6.0 (3)		1.82 (0.46–7.24)	0.39^d^
48–72 h	4.2 (2)	0.0 (0)		NA	0.99^d^
Patients with data	48	50			—
Use of metoclopramide % (Number of patients)					
0–24 h	37.5 (18)	64.0 (32)		0.34 (0.15–0.78)	0.01^d^
24–48 h	18.8 (9)	40.0 (20)		0.34 (0.14–0.88)	0.03^d^
48–72 h	12.5 (6)	28.0 (14)		0.36 (0.13–1.07)	0.07^d^
Patients with data	48	50			—
VAS score for pain, mm, estimation (SE)					
0–24 h	49.8 (3.9)	64.6 (3.8)	14.8 (4.0–25.6)		<0.01^d^
24–48 h	50.5 (3.9)	55.7 (3.8)	5.3 (−5.5–16.0)		0.34^d^
48–72 h	46.1 (3.9)	56.0 (3.8)	9.9 (−0.9–20.7)		0.07^d^
Patients with data	48	50			
PCA requirements during 72 h after surgery, times, median (interquartile range)					
	44.5 (14.5–88.0)	46.0 [8.0–85.5]			0.92^c^
Patients with data	47	50			
Cumulative fentanyl dose within 72 h, μg kg^−1^, mean (SD)					
	24.6 (13.8)	22.7 (14.0)	−1.8 (−7.4–3.8)		0.52^a^
Patients with data	47	50			
Postoperative blood loss within 24 h, ml, mean (SD)					
	368 (240)	380 (295)	11.3 (−96.9–119.5)		0.83^a^
Patients with data	48	50			
Presence of SSI within one month, % (Number of patients)					
	0.0 (0)	0.0 (0)		0.54^b^	
Patients with data	48	50		—	

^a^The Student’s t-test, ^b^Fisher’s exact test, ^c^Mann-Whitney *U* test or ^d^Generalized estimating equation analysis were used. CI, confidence interval; NA, not applicable; PCA, patient-controlled analgesia; PONV, postoperative nausea and vomiting; RR, relative risk; SD, standard deviation; SE, standard error; SSI, surgical site infection; VAS, visual analogue scale.

Significantly fewer patients in the dexamethasone group required metoclopramide during the first and second 24-h periods than those in the control group (RR 0.34, 95% CI 0.15–0.78 and RR 0.34, 95% CI 0.14–0.88; P < 0.01 and P = 0.03, respectively). The estimation of VAS score for nausea was significantly lower in the dexamethasone group than in the control group during the first 24 h postoperatively (difference in estimation 16.8 mm, 95% CI 4.6–29.0 mm, P = < 0.01), but not thereafter. There was no significant difference in the incidence of vomiting between the two groups during the study period. No difference was found in the beginning of the diet (1.1 postoperative day in the dexamethasone group and 1.2 postoperative day in the control group, p = 0.29).

Although PCA requirements and the cumulative dose of fentanyl administered in the 72 h after surgery were similar between the two groups, the mean VAS score for pain during the first 24 h postoperatively was significantly lower in the dexamethasone group than in the control group (difference in estimation 14.8 mm, 95% CI 4.0–25.6 mm, P < 0.01). In addition, the total dose of patient-requested flurbiprofen administered during the first 72 h after surgery was significantly reduced in the dexamethasone group (45.8 mg, 95% CI: 23.9–67.8 mg) than in the control group (117.0 mg, 95% CI: 75.9–158.1 mg) (p = 0.021). Five (10.0%) patients in the control group and four (8.3%) in the dexamethasone group discontinued intravenous PCA within 72 h because of severe PONV (Table 3). Intravenous PCA was terminated within 72 h in one (2.0%) patient in the control group and five (10.4%) in the dexamethasone group by the attending orthopaedic surgeon on the grounds that further pain relief was unnecessary. The amount of blood loss in the 24 h following surgery was not different between the two groups and no surgical site infections were reported during the month after surgery.

**Table 3:** Discontinuation of intravenous PCA within 72 h and reasons for discontinuation.

	Control (n = 50)	Dexamethasone (n = 48)	RR (95% CI)	P-value^c^
**Discontinuation of IV PCA**				
Total	6 (12.0)	10 (20.8)	1.1 (0.9–1.3)	0.28
**Reasons for discontinuation**				
Severe PONV	5 (10.0)	4 (8.3)	1.0 (0.9–1.1)	1.00
Reduced pain	1 (2.0)	5 (10.4)	1.1 (1.0–1.2)	0.11
PCA ineffective	0 (0.0)	1 (2.1)	1.0 (1.0–1.1)	0.49

^c^Fisher’s exact test was used. Values are shown as the number (%). RR, relative risk; CI, confidence interval; IV, intravenous; PCA, patient-controlled analgesia; PONV, postoperative nausea and vomiting.

## Discussion

In this randomized, single-centre, double-blind, prospective, placebo-controlled trial targeted to children and adolescents, a single dose of dexamethasone 0.15 mg/kg administered at induction of anaesthesia reduced the incidence and severity of PONV as well as on-demand use of metoclopramide after posterior correction and spinal fusion surgery for AIS. Further, this dose of dexamethasone improved early postoperative pain scores without an increase in adverse events. To our knowledge, this is the first study to assess the prophylactic effect of dexamethasone on PONV in paediatric patients undergoing a highly invasive surgical procedure that requires intravenous PCA for postoperative pain management.

Intravenous PCA with opioids is generally used for management of postoperative pain in patients with AIS who undergo scoliosis correction surgery, but is often accompanied by opioid-related side effects, such as PONV^9^. These patients are at particularly high risk of PONV because of the high proportion of girls^10^ and the long operating time, which are considered as major risk factors for PONV in paediatric patients^11^. More than 80% of the patients in this study who did not receive dexamethasone developed PONV, as reported previously^12,13^. Patients who experience PONV on PCA often refuse to continue bolus doses for pain relief, so adequate pain control relies on prevention of PONV, particularly in patients known to have multiple risk factors for PONV.

Dexamethasone is a synthetic glucocorticoid with anti-inflammatory and immunosuppressant properties. Although the mechanism of action remains unclear, dexamethasone is one of the agents most commonly used to prevent PONV^14^. The prophylactic effect of dexamethasone on PONV has been demonstrated in adults undergoing a wide range of surgical procedures^14,15^. In paediatric patients, it has been reported that dexamethasone 0.05–1.0 mg/kg administered at induction of anaesthesia reduced the incidence of PONV in the 24 h following common paediatric surgical procedures, such as tonsillectomy, correction of strabismus, and inguinal hernia repair^6,7,16^. A multicentre, multinational survey demonstrated that intravenous antiemetic prophylaxis is common practice for paediatric scoliosis surgery^8^. However, the prophylactic effect of dexamethasone on PONV in young patients undergoing highly invasive surgical procedures that require opioid-based PCA has not been clearly established. Dexamethasone has a long biological half-life (36–72 h)^17^, so may be particularly suitable for prevention of prolonged PONV caused by opioid-based PCA. In the present study, dexamethasone at a dose of 0.15 mg/kg, which is recommended in the latest guidelines^5^, reduced the overall incidence of PONV by 26% during the 72 h after surgery when compared with placebo. Moreover, it decreased the incidence of PONV and use of metoclopramide during the 0–24 h and 24–48 h postoperatively and the severity of nausea in the first 24 h after surgery. However, the incidence of vomiting and discontinuation of intravenous PCA because of severe PONV was similar between the two groups, suggesting that dexamethasone is more effective for reducing mild nausea than severe nausea or vomiting.

In addition to decreasing PONV, dexamethasone has been shown to reduce postoperative pain after several surgical procedures in both adults and children^18,19^. In the present study, the pain score during the first 24 h after surgery was significantly lower in the dexamethasone group than in the control group. Of note, in approximately 10% of the patients who received dexamethasone, intravenous PCA was terminated within 72 h after surgery because the attending orthopaedic surgeon considered that pain relief was no longer necessary. Use of multimodal analgesia and early discontinuation of intravenous PCA has been reported to reduce the length of hospital stay without an increase in pain scores^20^, so the analgesic properties of dexamethasone could also be of economic benefit in the perioperative management of patients undergoing posterior correction and spinal fusion surgery for AIS.

There are several limitations that should be borne in mind when interpreting the findings of this study. First, our sample size was not calculated to show differences in the secondary outcomes. Thus, it remains to be clarified whether dexamethasone 0.15 mg/kg reduces the incidence of PONV and use of metoclopramide in the late postoperative period (48–72 h). Second, the sample size was not large enough to determine the frequency of rare complications known to be associated with dexamethasone, such as hyperglycaemia and infection. We found no evidence of surgical site infection in this study. Although we did not examine the blood glucose level, it may be transiently elevated by a single dose of dexamethasone^21,22^. A recent meta-analysis and a large clinical trial did not find an increased risk of surgical site infection in patients with or without diabetes mellitus undergoing non-cardiac surgery who received a single dose of dexamethasone for PONV prophylaxis^23,24^. However, these trials and the studies involved in the meta-analysis did not aim to determine the safety of dexamethasone as a primary outcome. Therefore, a final judgment regarding the safety of dexamethasone should not be made until the results of an ongoing clinical trial of dexamethasone and surgical site infection (Perioperative Administration of Dexamethasone and Infection trial; ACTRN12614001226695) are available. Third, we did not determine whether the patients who received dexamethasone had an improved ability to ambulate and participate in physical therapy or whether they had a reduced length of hospital stay. Further investigations are needed to assess these potential additional benefits.

## Conclusion

Dexamethasone 0.15 mg/kg administered at the time of induction of anaesthesia reduced PONV and improved postoperative pain without increasing adverse events in paediatric patients undergoing scoliosis correction surgery. These results may support routine use of a prophylactic dose of dexamethasone in these patients.

## Supplementary information

CONSORT Checklist

Research Protocol

Raw data
