# Supplementary material for: Effects of prophylactic dexamethasone on postoperative nausea and vomiting in scoliosis correction surgery: a double-blind, randomized, placebo-controlled clinical trial
Source: Sci Rep. 2019 Feb 14;9:2119. doi: 10.1038/s41598-019-38764-8 (PMC6376007; doi:10.1038/s41598-019-38764-8)
Supplement: Supplementary file 2 — Research Protocol [file 41598_2019_38764_MOESM2_ESM.docx]

**Protocol title**

Prevention of postoperative nausea and vomiting with dexamethasone in children and adolescents undergoing posterior spinal fusion for adolescent idiopathic scoliosis: A randomized double-blind clinical trial

**Protocol identifying number**

This clinical trial was approved by the ethical committee at Keio University School of Medicine on 23 February 2015 (protocol number 20140395), and registered on the University hospital Medical Information Network (UMIN) Clinical Trials Registry on 19 March 2015 (UMIN000016847).

**Sponsor**

None

**Principal investigator**

Hiroyuki Seki, MD, PhD

Department of Anesthesiology, Keio University School of Medicine, 35 Shinanomachi, Shinjukuku, Tokyo, 160-8582, Japan

+81-3-3353-1211

**Name and address of the institutions involved in the research**

Keio University Hospital, 35 Shinanomachi, Shinjukuku, Tokyo, 160-8582, Japan

**Date**

30 January 2015

**Table of contents**

1. Project summary 3

2. Rationale & background information 4

3. Study goals and objectives 5

4. Study Design 5

5. Methodology 5

6. Flow diagram 7

7. Data management and statistical analysis 9

8. Expected Outcomes of the Study 9

9. Dissemination of Results and Publication Policy 9

10. Duration of the Project 10

11. Ethics 10

**1. Project summary**

<Rationale>

Dexamethasone is widely used for postoperative nausea and vomiting (PONV) prophylaxis, but its effect on PONV prevention in paediatric patients is validated only in short minor surgical procedures in which PONV may be induced by volatile anaesthetics or intraoperative opioids.

<Objectives>

In this study, we aimed to determine whether a single dose of dexamethasone reduces PONV in highly invasive surgeries that require patient-controlled analgesia (PCA), that can cause prolonged PONV.

<Methods>

On the day of surgery, the patients will be randomly assigned 1:1 to either of two study groups using a computer-generated random number table and receive intravenous dexamethasone 0.15 mg kg-1 in 5 ml of 0.9% normal saline (dexamethasone group; n=50) or volume-equivalent 0.9% normal saline (control group; n=50) at the time of anaesthesia induction.

All patients will receive total intravenous anaesthesia consisting of propofol, fentanyl and remifentanil. Fentanyl-based PCA will be provided as postoperative analgesia. Incidence of PONV in the 72 h postoperatively will be monitored as a primary outcome.

<Population>

Patients aged 10–19 years with a diagnosis of adolescent idiopathic scoliosis (AIS) and scheduled for posterior correction and fusion surgery at Keio University Hospital from May 2015 onwards are eligible for participation. The exclusion criteria are use of corticosteroids within the month before surgery, use of an antiemetic in the 24 h before surgery, and a contraindication to the study drug.

<Expected outcomes>

A single dose dexamethasone reduces 72 h-PONV in this patient population.

**2. Rationale & background information**

Scoliosis correction surgery has been described as the most invasive orthopaedic surgery performed in young persons^1^. This surgery is associated with severe postoperative pain that requires advanced pain management, which is typically opioid-based PCA. However, the management of opioid-related complications, in particular PONV, is still inadequate in the majority of patients^1^. Serious outcomes from anaesthesia are rare, but PONV is a major concern in surgical patients^2^. PONV can impair patient satisfaction, delay postoperative recovery, and increase medical costs^3^. PONV affects approximately one-third of surgical patients and up to 70% of high-risk patients^4^. For patients at high risk for PONV, prophylactic use of antiemetics including corticosteroids is recommended in international consensus guidelines^5^. However, the evidence for prophylactic use of dexamethasone in paediatric patients is based only on minor surgical procedures, such as tonsillectomy and strabismus surgery^6,7^, in which PONV may be induced by volatile anaesthetics or intraoperative opioids. Although the use of dexamethasone for PONV prophylaxis is common practice in paediatric scoliosis surgery^8^, the effect of dexamethasone on PONV prevention has not been validated in highly-invasive surgical procedures that require opioid-based PCA for postoperative analgesia, in which PONV can be prolonged by opioids.

**References**

1 Rullander, A. C., Jonsson, H., Lundstrom, M. & Lindh, V. Young people's experiences with scoliosis surgery: a survey of pain, nausea, and global satisfaction. Orthop Nurs 32, 327–333; quiz 334–325 (2013).

2 Macario, A., Weinger, M., Carney, S. & Kim, A. Which clinical anesthesia outcomes are important to avoid? The perspective of patients. Anesth Analg 89, 652–658 (1999).

3 Parra-Sanchez, I. et al. A time-motion economic analysis of postoperative nausea and vomiting in ambulatory surgery. Can J Anaesth 59, 366–375 (2012).

4 Gan, T. J. Postoperative nausea and vomiting--can it be eliminated? JAMA 287, 1233–1236 (2002).

5 Gan, T. J. et al. Consensus guidelines for the management of postoperative nausea and vomiting. Anesth Analg 118, 85–113 (2014).

6 Steward, D. L., Grisel, J. & Meinzen-Derr, J. Steroids for improving recovery following tonsillectomy in children. Cochrane Database Syst Rev, CD003997 (2011).

7 Madan, R. et al. Prophylactic dexamethasone for postoperative nausea and vomiting in pediatric strabismus surgery: a dose ranging and safety evaluation study. Anesth Analg 100, 1622–1626 (2005).

8 Palmer, G. M., Pirakalathanan, P. & Skinner, A. V. A multi-centre multi-national survey of anaesthetists regarding the range of anaesthetic and surgical practices for paediatric scoliosis surgery. Anaesth Intensive Care 38, 1077–1084 (2010).

**3. Study goals and objectives**

The goal of this study is to determine whether dexamethasone reduces PONV in children and adolescents undergoing highly invasive surgery, such as posterior correction and spinal fusion surgery for AIS.

The primary objective is the incidence of PONV in the 72 h after surgery. Nausea is defined as a subjective feeling of a desire to vomit without the presence of expulsive muscular movements. Vomiting is defined as the involuntary, forceful expulsion of the contents of stomach. Retching will be included as vomiting. The patient is considered to have nausea when the VAS score for nausea is more than 0 and to have PONV if nausea or vomiting occurs or rescue metoclopramide is administered.

The secondary objectives include the incidence of PONV, vomiting, use of rescue metoclopramide, VAS scores for nausea and pain in the 0–24, 24–48, and 48–72 h after surgery, number of PCA doses requested by the patient and total amount of fentanyl administered in the 72 h postoperatively, amount of blood loss in the 24 hours after surgery, and incidence of surgical site infection in the month following surgery.

**4. Study Design**

This is a randomized, single-centre, double-blind, prospective, placebo-controlled clinical trial.

Patients aged 10–19 years with a diagnosis of AIS and scheduled for posterior correction and fusion surgery at Keio University Hospital from May 2015 onwards are eligible for participation. The exclusion criteria are use of corticosteroids within the month before surgery, use of antiemetics in the 24 h before surgery, and a contraindication to the study drug.

Expected duration of this study is 5 years.

**5. Methodology**

On the day of surgery, the patients will be randomly assigned 1:1 to either of the two study groups using a computer-generated random number table. Randomization will be performed by an anaesthesiologist who is not involved in the trial. The random number table will be kept by the anaesthesiologist and unblinded by a statistician who will not be involved in the study and will perform statistical analyses after patient recruitment is complete. Participants and their parents, the surgeon, anaesthesiologists, nurses, and the investigator who will collect the data will be blinded to study group allocation.

All patients receive intravenous propofol 2.5 mg kg-1, fentanyl 4 μg kg-1, and rocuronium 0.6 mg kg-1 for induction of anaesthesia. At this time, the patients also receive intravenous dexamethasone 0.15 mg kg-1 in 5 ml of 0.9% normal saline (dexamethasone group; n=50) or volume-equivalent 0.9% normal saline (control group; n=50). The study drugs will be prepared by a pharmacist who is not involved in the study. The patients, parents, and health care providers including the anaesthesiologists and nurses in the operating room, intensive care unit, and the ward will remain unaware of the group allocation for each subject. After tracheal intubation, anaesthesia will be maintained with propofol (adjusted to maintain a bispectral index of 40–60), intermittent administration of fentanyl, and infusion of remifentanil. Motor evoked potentials will be monitored after administration of sugammadex with the patient in the prone position. After emergence from anaesthesia, the trachea will be extubated, and the patient will be transferred to the intensive care unit.

Postoperative analgesia will comprise intravenous PCA with fentanyl (0.2 μg kg-1 h-1 as background infusion and 0.4 μg kg-1 as a bolus dose, with a lockout interval of 10 min), infusion of ketamine 0.1 mg kg-1 h-1, and administration of a 25-mg diclofenac sodium suppository every 6 h. In addition, intravenous flurbiprofen 50 mg will be administered upon patients’ request. Metoclopramide 10 mg will be administered intravenously to treat PONV on patient request. Intravenous PCA will be continued for at least 3 days postoperatively unless otherwise specified.

Before surgery, the patients will be provided with instructions regarding use of the PCA device and the visual analogue scale (VAS) for nausea and pain (0–100 mm). At 24, 48, and 72 h after surgery, all patients will be asked by trained ward nurses, who will be blinded to study group allocation, to complete a VAS sheet describing the worst levels of nausea and pain experienced during the preceding interval.

If the patient and/or the parents withdraw consent before anaesthesia induction, the patient will be excluded from the study, i.e., no study medications will be given, and no data will be collected. If serious adverse events that are considered to be associated with the use of dexamethasone occur, the code can be broken by the anaesthesiologist who randomized the patients, and discussion will take place on whether to stop the trial.

Data to be collected are described below.

1. Patient characteristics and intraoperative variable

Age, gender, height, Cobb angle, number of levels fused, duration of surgery, duration of anaesthesia, doses of intraoperative fentanyl and remifentanil, amount of intraoperative blood loss.

2. Primary and secondary outcomes

Incidence of 0-72 h PONV, incidence of PONV, VAS score for nausea and pain, incidence of vomiting, and the use of rescue-metoclopramide in the 0-24, 24-48, 48-72 h after surgery, PCA requirements and cumulative fentanyl dose within 72 h after surgery, amount of postoperative blood loss within 24 h, and presence of surgical site infection within one month.

**6. Flow diagram**

Total N: Obtain informed consent. Screen potential subjects by inclusion and exclusion criteria; obtain history, document

Randomize

All patients will receive intravenous propofol, fentanyl, and rocuronium for induction of anaesthesia.

At this time, the patients also receive intravenous dexamethasone 0.15 mg kg-1 in 5 ml of 0.9% normal saline or volume-equivalent 0.9% normal saline.

Anaesthesia

Induction

Anaesthesia will be maintained with propofol (adjusted to maintain a bispectral index of 40–60), intermittent administration of fentanyl, and infusion of remifentanil. Motor evoked potentials is plan to be monitored after administration of sugammadex with the patient in the prone position. After emergence from anaesthesia, the trachea will be extubated, and the patient will be transferred to the intensive care unit.

Surgery—

Recovery

All patients will be asked by trained ward nurses blinded to study group assignment to complete a VAS sheet describing the worst levels of nausea and pain experienced during the preceding interval.

24 hours

after surgery

---Continue to next Page

---Continue from previous page

48 hours

Same as previous time point

after surgery

Same as previous time point

72 hours

after surgery

Collect the data of PCA

day 3-7

Final Assessment

**7. Data Management and Statistical Analysis**

<Data management>

Randomization will be performed by an anaesthesiologist who is not involved in the trial using a computer-generated random number table. Participants and their parents, the surgeon, anaesthesiologists, nurses, and the investigator who will conduct follow-up postoperatively will be blinded to study group allocation.

Data will be collected and recorded on a case report form by anaesthesiologists who are not involved in this study. The case report form will be stored in a box that is key-locked in the office of the department of anaesthesiology, Keio university hospital. After completing patient follow-up, the data will be analysed by a statistician who is not involved in this study.

An audit by the ethics committee will be performed at an unknown timing.

<Statistical analysis>

A power analysis was performed using a power of 80% and an α of 0.05 (two-sided). Our retrospective observations showed that 26 (79%) of 33 patients developed PONV within the 72 h after surgery. Thus, we assumed that the incidence of PONV in the 72 h after surgery in the control group would be approximately 80%. We considered that a 30% reduction in incidence of PONV would be clinically relevant. The power analysis showed that 46 patients were needed in each study group. Fifty patients were enrolled in each group to allow for possible dropouts.

The data will be analysed based on the intention-to-treat population, i.e., all patients who are randomized, received the study drug, and underwent surgery. Categorical data will be presented as frequencies, and continuous data will be summarized as the mean (standard deviation) or median [interquartile range]. Continuous parametric and non-parametric data will be compared using the Student’s t-test and Mann-Whitney U test, respectively. Categorical data will be compared using Fisher’s exact test. For sensitivity analysis, the incidences of PONV and vomiting, frequency of use of rescue metoclopramide, and VAS scores for nausea and pain at each time-point will be estimated by the generalised linear mixed model to obtain point estimates and 95% confidence limits. The correlation structure will be assumed as Toeplitz, autoregressive, or compound-symmetry structures and will be used in order if convergence is not obtained. A P-value of <0.05 is considered statistically significant.

**8. Expected Outcomes of the Study**

This study will add evidence that dexamethasone can reduce PONV in children and adolescents undergoing not only minor, short surgical procedures, but also highly invasive surgery. Evidence in Japanese is needed to use dexamethasone for PONV prophylaxis in Japan as dexamethasone has not yet been approved in Japan, although it is being widely used worldwide.

**9. Dissemination of Results and Publication Policy**

In addition to presenting a summary of this review in an international scientific meeting, a paper will be submitted to a leading journal in this field.

**10. Duration of the Project**

Estimated duration of the project will be a total of 5 years (for recruiting patients: 2-3 years; for preparing manuscript and publishing: 1-2 years)

**11. Ethics**

This randomized, single-centre, double-blind, prospective, placebo-controlled clinical trial was approved by the ethical committee at Keio University School of Medicine on 23 February 2015 (protocol number 20140395) and registered on the University hospital Medical Information Network (UMIN) Clinical Trials Registry on 19 March 2015 (UMIN000016847). The study is carried out in accordance with the ethical standards of the Declaration of Helsinki of 1975.

Written informed consent will be obtained from the parents of the patients along with assent from the patients at the preoperative anaesthesia clinic or during the preoperative round.

To prepare for compensation for health damage for which causal relationship with this research cannot be denied, the researchers are applying for clinical research insurance.
